# Understanding the influence of parent-clinician communication on antibiotic prescribing for children with respiratory tract infections in primary care: a qualitative observational study using a conversation analysis approach

**DOI:** 10.1186/s12875-019-0993-9

**Published:** 2019-07-19

**Authors:** Christie Cabral, Jeremy Horwood, Jon Symonds, Jenny Ingram, Patricia J. Lucas, Niamh M. Redmond, Joe Kai, Alastair D. Hay, Rebecca K. Barnes

**Affiliations:** 1 Centre for Academic Primary Care, Population Health Sciences, Bristol Medical School, University of Bristol, 39 Whatley Road, Bristol, BS8 2PS UK; 2 Children and Families Research Centre, School for Policy Studies, University of Bristol, 8 Priory Road, Bristol, BS8 1TZ UK; 3 Centre for Academic Child Health, Population Health Sciences, Bristol Medical School, University of Bristol, Bristol, BS8 1NU UK; 4 Centre for Health & Social Care, School for Policy Studies, University of Bristol, 8 Priory Road, Bristol, BS8 1TZ UK; 5 Division of Primary Care, School of Medicine, University of Nottingham, Nottingham, NG7 2UH UK

**Keywords:** Primary care, Antibiotics, Children, Parents, Communications, Conversation analysis, Respiratory tract infections

## Abstract

**Background:**

Acute respiratory tract infections (RTI) in children are a common reason for antibiotic prescribing. Clinicians’ prescribing decisions are influenced by perceived parental expectations for antibiotics, however there is evidence that parents actually prefer to avoid antibiotics. This study aimed to investigate the influence of parent-clinician communication on antibiotic prescribing for RTI in children in England.

**Methods:**

A mixed methods analysis of videoed primary care consultations for children (under 12 years) with acute cough and RTI. Consultations were video-recorded in six general practices in southern England, selected for socio-economic diversity. 56 recordings were transcribed in detail and a subset of recordings and transcripts used to develop a comprehensive interaction-based coding scheme. The scheme was used to examine communication practices between parents and clinicians and how these related to antibiotic or non-antibiotic treatment strategies.

**Results:**

Parents’ communication rarely implied an expectation for antibiotics, some explicitly offering a possible viral diagnosis. Clinicians mostly gave, or implied, a viral diagnosis and mainly recommended non-antibiotic treatment strategies. In the minority of cases where parents’ communication behaviours implied they may be seeking antibiotic treatment, antibiotics were not usually prescribed. Where clinicians did prescribe antibiotics, they voiced concern about symptoms or signs, including chest pain, discoloured phlegm, prolonged fever, abnormal chest sounds, or pink /bulging ear drums.

**Conclusions:**

We found little evidence of a relationship between parents’ communication behaviours and antibiotic prescribing. Rather, where antibiotics were prescribed, this was associated with clinicians’ expressed concerns regarding symptoms and signs.

## Background

High rates of antibiotic prescribing have led to increased antimicrobial resistance (AMR) [1, 2] and higher rates of morbidity and mortality from resistant infections [3]. Understanding the drivers of antibiotic prescribing has therefore become a key priority for health research globally [4, 5]. Acute respiratory tract infections (RTI) in children are a common reason for antibiotic prescribing [6]. There are multiple influences on the decision to prescribe antibiotics, including clinicians’ interpretation of the clinical signs [7, 8], concern for the safety of the child [9, 10] and perceived pressure from parents [11, 12].

Another key influence on whether antibiotics are prescribed is communication between clinicians and patients or carers [13, 14]. We know that parents rarely make explicit requests for antibiotic treatment for their children [15, 16]. However, parents may suggest a possible diagnosis (e.g. “strep throat”) or symptoms (e.g. “chesty cough”) that could indicate a need for antibiotic treatment and lead clinicians to perceive parental antibiotic expectations [15, 17], which in turn is associated with higher antibiotic prescribing [12, 18].

Studies that found an association between antibiotic prescribing and clinician perception of parental expectations, also showed parents’ actual expectations of antibiotic treatment were not predictive of antibiotic prescribing [12]. When parents offer a possible diagnosis or identify concerning symptoms, they may be seeking reassurance that the consultation is justified or that their child is not seriously ill (rather than indirectly requesting antibiotics) [18]. Parents and clinicians often talk at cross purposes about the seriousness of the child’s illness and this miscommunication may contribute to over-prescription of antibiotics [19], with studies of parental views consistently finding a preference for no treatment for minor infections [10, 20].

Our systematic review found few studies examining the relationship between parent-clinician communication and antibiotic prescribing, with none in a UK context and most undertaken over a decade ago [19]. In the USA (a high antibiotic prescribing context), the relationship between actual parental expectations, clinician-perceived expectations and inappropriate antibiotic prescribing has been previously explored using survey data [12] and observational data from video-recordings of clinical encounters [12, 21]. Conversation analytic (CA) methods can be applied to detailed transcripts of recordings to identify the underlying rules and norms that guide key communication activities or tasks (such as soliciting patients’ problems, diagnosing and prescribing), and how these norms can affect outcomes [22]. Using CA methods, Stivers identified two key parental communication behaviours that occur in different phases of paediatric primary care consultations and appear to influence clinician perception of parental antibiotic expectations: firstly what is said at the beginning of the consultation and secondly what is said following treatment recomendation [21, 23]. At the beginning of the consultation, Stivers distinguishes between communications which lead clinicians to perceive treatment expectations (when parents suggest a “candidate diagnosis”, or “implied candidate diagnosis” using symptoms), and those clinicians perceive as being free of treatment expectations (a “symptoms only” presentation) [21, 23]. After the delivery of treatment recommendations by clinicians, Stivers distinguishes between clear acceptance and responses indicating “interactional resistance” (silence, a treatment obstacle or a question) [21]. These findings are supported by a Finnish CA study in the same context that examined parent-clinician communication and found that similar parental communication patterns influenced clinicians’ communication. However this study did not examine the influence on antibiotic prescribing (antibiotic prescribing rates were much lower than reported in the USA study) [24, 25].

Our study aims to build on this earlier work by examining whether these same parental communication patterns can be found in primary care consultations for children with RTI in the UK, and whether they are associated with antibiotic prescribing.

## Method

The data consisted of primary care consultations for children with RTI that were recorded between May and December 2013. These recordings were made for a previous study and the data collection methods are described in detail elsewhere [26]. The previous study recorded 60 complete consultations, 56 video recorded and 4 audio only, the analysis for this study was conducted on the 56 consultations that were video recorded. Parents and children were recruited in six General Practices in the South-West of England selected to represent a range of neighbourhoods including both deprived and affluent. Thirteen clinicians agreed to participate, including nine General Practitioners (GPs), three Nurse Practitioners (NPs) and one Physician’s Assistant (PA). Eighty-three parents of children aged 3 months to 12 years who presented with an acute RTI with a cough were invited to participate, 72% agreed and provided written informed consent. 13 consultations included non-native speakers. The study design was guided by a Public Patient Involvement group of local parents.

Our mixed methods CA approach combined qualitative CA-grounded coding of the recorded consultations with subsequent descriptive quantitative analysis [22, 27]. In preparation for the initial coding, the recordings were transcribed in detail according to standard Jeffersonian conventions [28]. A comprehensive coding scheme was developed that spanned parent, child and clinician activities across the whole consultation. Initial codes for each of the consultation activity phases – opening, problem presentation, history-taking, physical examination, diagnosis, treatment planning and closing - were developed deductively by CC, RB and JS, drawing on findings from published CA studies of routine primary care consultations [15, 21, 29, 30], and inductively by working with a 15% subset of our transcripts and recordings. This subset included a range of different practice settings, clinicians and outcomes. CC, RB and JS engaged in a series of CA data sessions each focused on a different activity phase. These involved repeatedly watching relevant extracts from the videos and working line-by-line through the transcripts to identify common patterns in the communication behaviours relating to key activities or tasks. Communication behaviours in our data not previously studied, for example safety-netting practices, were assigned new interaction codes.

A first version of the coding scheme was then drafted for testing. The scheme included a codebook with definitions of the codes, coding criteria and clear exemplars. Further focused meetings were then held to test these codes across the original subsample. Consequently, codes were refined or dropped and/or new codes were identified. A randomly selected further 15% subsample were then independently double coded by JS and RB and further amendments and revisions made based on discussions between JS, RB and CC. In the same way, a second and third 15% subsample were independently coded until consensus was reached on the coding scheme. This Four of these types were identified by previous checking and redefining the communication phenomena captured by the code labels ensured a robust coding framework. One researcher (JS) then applied this coding scheme to the whole data set. The frequencies of coded communication behaviours were then examined across the dataset and in relation to treatment recommendations.

This paper reports on the distribution of codes for parent problem presentation, diagnoses delivered, and treatments recommended by clinicians and the relationship between these.

## Results

The 56 video-recorded consultations were for 60 children with RTI (in four consultations, two child patients were present), 78% of children were under 5 years and 67% were male (Table 1). The sample of patients and parents was diverse, from both deprived and affluent neighbourhoods and 36% of parents were of non-white ethnicity (Table 1). The 13 participating clinicians had varied experience from < 5 to > 15 years in primary care and included GPs, NPs and a Physician Assistant (Table 1). The consultation duration ranged from 4 min and 12 s to 28 min and 40 s, with an average of 10 min and 45 s.

**Table 1:** Sample description

Child (*n* = 60) & Parent (*n* = 56)		Clinician (*n* = 13)	
Child gender		Clinical role	
Female	20	Doctor (GP)	9
Male	40	Nurse (NP)	3
Child age		Physician’s Assisstant (PA)	1
< 2 years	21	Clinician experience	
2-4 years	26	< 5 years	4
5–12 years	13	5–15 years	4
Parent ethnicity		> 15 years	5
White British	36		
Eastern European	7		
Black	9		
Mixed	2		
Asian	2		
Home neighbourhood deprivation level			
(most deprived) 1	13		
2	18		
3	11		
4	11		
(most affluent) 5	3		

### Parent problem presentation

Five different types of problem presentation by parents were identified and coded (Table 2). Four of these types were identified by previous CA studies but one type ‘viral candidate diagnosis’ is identified and coded for the first time in this study. In the majority (41, 73%), the parents’ problem presentations did not imply an expectation of antibiotic treatment. These included ‘symptom only’ presentations, ‘viral candidate diagnosis’ (explicit or implied) and ‘candidate explanations’ for concerning symptoms such as vomiting. In the remaining cases (27%) parents either suggested candidate diagnoses (e.g. “chest infection”) or cited symptoms (e.g. “phlegmy … on the chest”) that a clinician may understand to imply a possible need or expectation for antibiotic treatment.

**Table 2:** Parent problem presentation

Problem Presentation type	Frequency (%)	Example
Symptoms only	23 (40%)	*“she’s had a cold and a cough, er, for about a couple of weeks now. (…*) *the cough’s sort of getting worse. She’s still got a bit of a runny nose. Erm, and it’s sort of waking her up at night. And I just really want some guidance.”* (P52: mother)
Viral candidate diagnosis	11 (20%)	“*it looked like it was a viral infection”* (P01: father)
Candidate explanations	7 (13%)	*“I think sometimes he’s just coughing so much that he’s ending up being sick”* (P03: mother)
Antibiotic implicative candidate diagnoses	7 (13%)	“*she’s had a really bad chest infection*” (P13: father)
Antibiotic implicative symptoms only	8 (14%)	“*she’s now sounding really phlegmy, and right on the chest*” (P56: mother)

### Clinician diagnoses and treatment recommendations

A viral diagnosis was the most common and delivered explicitly using the term “virus” or “viral” in 37 (66%) consultations and implied in a further nine (16%) consultations by terms like “cold’” or described as an unproblematic illness (Table 3). In six (11%) cases a diagnosis of a chest or ear “infection” was given, and one child was diagnosed with pneumonia. In three (5%) cases there was no explicit diagnosis, but specific symptoms were identified as concerning and linked to the prescription of antibiotics, implying the diagnosis of a possible bacterial infection.

**Table 3:** Types of diagnosis

Diagnosis	Frequency (%)	Example
Viral diagnosis (explicit)	37 (66%)	“*So it all looks as if he’s got a virus*” (C212: GP)
Viral diagnosis (implied)	9 (16%)	“*a usual cough*” (C218: GP) “*there doesn’t seem to be anything sinister happening at the moment*” (C202: PA)
‘Infection’ diagnosis	6 (11%)	“*this probably looks more like she has got a bit of a chest infection rather than a viral cold*” (C220: NP)
Pneumonia diagnosis	1 (2%)	“*if a child complains of chest pain it is a pneumonia until you can prove otherwise.*” (C19: GP)
No diagnosis but bacterial infection implied by antibiotic prescription linked to specific symptoms	3 (5%)	“*I think if the phlegm was yellowy, it’s probably worth using an antibiotic*” (C213: GP) “*she’s had a temperature for now, for over a week coming down. That’s quite a long time to be having a temperature*” (C224: NP)

The most common treatment recommendation was home care (Table 4). This varied widely from a simple watch and wait strategy to detailed instructions about how to manage the symptoms. Antibiotics were prescribed in 12 (21%) cases, eight (14%) for immediate consumption and four (7%) delayed prescriptions. Two children had both antibiotics and steroids prescribed, in both cases they were given a primary diagnosis of an infection, for which antibiotics were prescribed and the steroids (tablets or inhalers) were prescribed as additional symptom relief. Steroids alone were prescribed for 10 (18%) cases as symptom relief treatment. In three (5%) cases, over-the-counter (OTC) medicines (paracetamol and non-steroidal anti-inflammatory drugs and in one case with cough medicine in addition) were prescribed in response to direct requests for these from parents.

**Table 4:** Treatment recommendation

Treatment recommendation	Frequency (%)	Verbatim Example
Home care advice only	31 (55%)	“*I think let’s see how he goes*” (C220: NP) *“lots of rest, lots of fluid. Calpol [child paracetamol] if he needs it … Make sure he eats and drinks plenty. He can probably go to school now*” (C214: GP)
Antibiotics (immediate)	8 (14%)	*“Well, she seems really well. … But, because I can hear that little noise in her chest, I’m going to give her some antibiotics because it sounds like there’s a little infection in there.” (C219: NP)* “*I think if the phlegm was yellowy, it’s probably worth using an antibiotic*” (C213: GP) “*I think he has an infection in his ear. And that might be why he’s getting the temperatures and being sick. … I think that will improve with the antibiotics for his ear*.” (C205: GP)
Antibiotics (delayed)	4 (7%)	“*by Monday, if she’s still having temperatures, even if she gets no more unwell in herself, then that’s gone on longer than I would expect with a viral thing, and I would want her to have antibiotics at that point.*” (C205: GP) *“his right ear drum is a lot more swollen and red that the left okay, which I think is where the infection’s stemming from … I wouldn’t give him antibiotics at the moment but I will give you the prescription … by tomorrow it will be more than 3 days, I would hope that … his temperature has started to come down. If not, start the antibiotics.”* (C203: GP)
Steroids only	10 (18%)	“*His lungs at the bottom … are slightly [wheezy] sounding, which is normally what we hear with a viral chest infection … I’m not really sure that he’s going to benefit … on antibiotics, but he might benefit from a little bit of inhaler, just to try and loosen the chest slightly*.” (C207: GP)
OTC prescribed	3 (5%)	*Mother: “could I possibly have a prescription for Calpol?* *GP: Yeah, no problem. (C201: GP)* *Mother: Is that alright? Just ‘cos … I haven’t brought my wallet with me this – you know.” (P11: mother)*

In 11 of the 12 cases where antibiotics were prescribed, the clinician linked this decision to specific clinical observations (Table 4). Immediate antibiotics were linked to sounds heard during the chest examination, pain in the chest, yellow phlegm and ear infection. Delayed antibiotics were linked to fever of long duration. There was one case where delayed antibiotics appeared to be issued in response to parental interactional resistance to a non-antibiotic treatment recommendation (see Table 6).

**Table 5:** Relationship between problem presentations and outcome

		Treatment Recommendation (N and % of consultations in each presentation category)		
Parent problem presentation type	*N*	Home care advice/OTC	Antibiotic prescribed	Steroids prescribed
Symptoms only	23	12 (52%)	7 (30%)	4 (17%)
Viral candidate diagnosis	11	6 (55%)	2 (18%)	4 (17%)
Candidate explanations	7	6 (86%)	1 (14%)	1 (14%)
Antibiotic implicative candidate diagnosis	7	5 (71%)	1 (14%)	1 (14%)
Antibiotic implicative symptoms only	8	5 (63%)	1 (13%)	2 (25%)

### Relationships between forms of communication and treatment decisions

Parents’ problem presentations did not appear to be associated with antibiotic treatment decisions (Table 5). Antibiotics were most often prescribed following ‘symptoms only’ and ‘viral candidate diagnoses’ presentations. In the 15 consultations in which parents presented a candidate diagnosis (e.g. “chest infection”) or symptom (e.g. “it is on the chest”) that implied an antibiotic may be appropriate, antibiotics were prescribed twice and steroids three times (Table 5). Steroids were prescribed more often for ‘symptoms only’ and ‘viral candidate diagnosis’ presentation cases.

**Table 6:** Parent immediate response to clinician treatment recommendations

Parent response	Total number (%)	Example
Acknowledgement	43 (77%)	“*I don’t think we need to do anything. I think you’re doing all the right things in terms of lots of rest, lots of fluid. Calpol if he needs it. But I don’t think – if he’s like this I wouldn’t worry about*. ”(C214: GP) “Right, okay.” (P23: father) “i*t’s probably worth starting a course of antibiotics*.” (C214: GP) “*Okay*.” (P54: mother) “*I’ll give you just the puffer as a precaution for the next week, alright.”* (C215: GP) “*Yeah*.” (P26: mother)
Active Acceptance	3 (5%)	“*I don’t think she needs antibiotics today*” (C224: NP) “*No, … no and to be honest I’d rather she didn’t*” (P65: mother)
Interactional resistance	6 (11%)	GP: “*She does have an ear infection …*. *usually they will recover within 5 days, and that’s not sped up by taking antibiotics. … I think [give it] 5 days if she doesn’t become unwell in herself.”* *Father: “So if she does basically um, after 5 days, just then, what, just go to the er local um pharmacy and get some um*?” *GP: “Well after – what I’ll do is I’ll give you a prescription now for some antibiotics.” (C205:GP)* *Father: “Okay.” (P13: father)* *“if we treat the ear infection* [with antibiotics]*, she’ll feel a bit better in herself … The cough should, sort of, limit on its own. However, if she’s worse, you think the cough’s worse, or you’re worried about her breathing, then come back.” (C220: NP)* *“Yeah. Is there, erm, anything that kind of helps with her [cough]?*” (P40: mother) *“Her chest in completely clear. But actually, oddly, the paeds A&E consultants will say that if a child complains of chest pain … it is a pneumonia until you can prove otherwise.* *… I would actually say in this case she probably would benefit from a 5 day course of antibiotics.”* (C207: GP) *“Because she’s complaining about pain?”* (P19: mother)

The most common response to any treatment recommendation was a simple acknowledgement, which was sometimes stated verbally and sometimes communicated non-verbally with a nod. In 43 (77%) cases, parents acknowledged the clinicians’ primary treatment recommendation by either nodding or saying just one or two words; in three cases (5%) parents actively accepted the recommendation; in six cases (11%) parents displayed active ‘interactional resistance’; and in the remaining four (7%) cases there was no clear response to the treatment recommendation (Table 6). After receiving an acknowledgement or acceptance from a parent, the clinician usually continued almost immediately to give further home care and safety-netting advice.

In the six cases where parents displayed active ‘interactional resistance’, parental responses to the treatment recommendation came in the form of a question. In one case the parent (P13) was responding to a ‘no-antibiotic’ treatment recommendation; in one case the parent (P19) was responding to a recommendation for antibiotic treatment; and in the remaining four cases, parents responding to home care recommendations. In the one case of ‘interactional resistance’ to a ‘no-antibiotic’ treatment recommendation, the parent questioned what to do after the recommended five-day watch and wait period and informed the GP that accessing same day appointments was difficult; a delayed prescription was subsequently issued for use if needed after 5 days (see Table 6). In the case of a parent resisting a recommendation for antibiotic treatment, parents asked a clarifying question about the reason the clinician felt antibiotics were needed (P19) (see Table 6). In the four cases of ‘interactional resistance’ to home care recommendations, parents were asking for more detailed advice on effective symptomatic treatments.

## Discussion

### Summary

In these consultations, parental communication behaviours did not appear to be linked with antibiotic prescribing. Where antibiotic-indicative candidate diagnoses were presented by parents, this was not associated with higher rates of antibiotic prescription. The most common problem presentation was “symptoms only”, which imply parents are seeking medical evaluation, rather than a particular diagnosis and treatment [21, 23]. In 50 of the 56 consultations, parents responded with acknowledgement or clear acceptance of the treatment recommendations, including parents who had used antibiotic-indicative presentations. Parents responded with active resistance in only six consultations. Clinicians recommended non-antibiotic treatment in the majority of cases and when they did prescribe antibiotics, it was usually linked to a symptom or sign about which they had expressed concern.

### Comparison with existing literature

This study found that fewer parents used antibiotic implicative candidate diagnoses compared with the earlier US-based study [15], but the frequencies recorded here are similar to those reported by a recent Finnish study [24]. Our study was carried out in a time when awareness of over prescription of antibiotics was (and is) high and in a different health system context (UK) and where antibiotic prescribing is lower than in the US. The US study was also conducted in predominantly mid to high income populations, whereas our study population encompassed the full range of income levels. These contextual differences may account for the different findings. Our data provide evidence that in the current UK context, parents’ communication behaviours may not be a major influence on antibiotic prescribing in primary care (in hours) for RTI in children.

When parents offer a candidate diagnosis, they are both displaying their own expertise and justifying the need for a consultation. Parents’ communication behaviours within these consultations draw on their knowledge and experience as a way of furthering a shared understanding with the clinician [16, 19, 25]. When parents consult for an RTI in a child the use of a candidate diagnosis can be a means of directing the clinician’s attention to a key concern [31]. For example, using the term ‘chest infection’ may be a way of ensuring this possibility is evaluated by the clinician, and these concerns may be assuaged by a credible evaluation [20]. This study is the first to record the use of viral candidate diagnoses by parents, which may indicate that parents are using this form of problem presentation to indicate they are not seeking antibiotics.

The CA term ‘interactional resistance’ is often interpreted as parents opposing a treatment recommendation [32]. However, this term is meant to describe anything other than clear acceptance (e.g. the parent responds with silence, a minimal acknowledgement, a treatment obstacle or a question), in other words ‘resisting’ the norm or expected response of acceptance. The interactional consequence of interactional resistance is that progress towards next steps such as closing the consultation is delayed [21, 33]. In our study, as in previous work [25], we can see that parents are seeking to understand rather than challenge. Time pressure may be relevant here as clinicians feel the need for consultations to be completed quickly [8, 10], and may resort to prescribing as a way to end the discussion. Pressure to explain may be experienced as pressure to prescribe.

The antibiotic prescribing observed in this study was overtly linked to symptoms of concern observed by clinicians. There is variation between clinicians in the clinical symptoms and signs used to decide when to prescribe antibiotics [8, 34]. This may be because some clinicians are taking a more cautious approach, prescribing ‘just in case’ if they feel there is any risk of negative consequences to the child’s health or themselves (medico-legal) of not prescribing [9, 10].

### Strengths and limitations

This was the first study in the UK to use videos of real consultations to explore associations between parental communication and antibiotic prescribing for children with RTIs. Although our study was smaller than previous work in the US (*n* = 360), it was similar to the Finnish study (*n* = 70) and our study population was more diverse than previous studies, which drew patients from predominantly mid to high income populations. This sample provides for a robust qualitative and descriptive analysis but limited the inferential statistical analysis possible. We recruited a diverse range of families from deprived and affluent neighbourhoods and included a large proportion of non-White British ethnicities, although few Asian. In five of the six practices, clinicians had no influence over which consultations were recorded. One practice triaged all patients requesting same day appointments and, while clinician selection cannot be ruled out, these 10 consultations had similar characteristics to the others. The use of oral steroids in 12 cases implies a significant proportion had a relatively severe illness and we captured a representative range of cases. Clinicians with a range of professional experience were recruited, although, since they had to ‘opt-in’ to the study, participating clinicians may differ from those who did not take part.

## Conclusion

Antibiotic prescribing for children with RTI appears to be influenced mainly by clinicians’ reported interpretation of the symptoms and signs, rather than parental communication behaviours. Parental communication behaviours indicating that they may be seeking antibiotics appeared in few consultations and there was no clear relationship between parental communication behaviours and antibiotic prescribing. If antibiotic prescribing for RTI in children is to be improved, then interventions need a broader focus to include issues that may play a larger role, such as clinical uncertainty, safety concerns and external pressures on clinicians [9, 10, 35].

## Data Availability

The whole datasets generated and/or analysed during the current study are not publicly available because the data are videos of medical consultations and it was not possible to anonymise these (face of child patient, parents and clinicians are visible). However anonymised transcripts of the video encounters are available from the corresponding author on reasonable request.

## Abbreviations

AMR: Antimicrobial resistance
CA: Conversation analytic
GP: General Practitioner
NP: Nurse Practitioners
OTC: Over-the-counter
PA: Physician’s Associate
RTI: Respiratory tract infections
